# Health-related quality of life and associated factors among people living with human immunodeficiency virus on highly active antiretroviral therapy in North East Ethiopia: Cross-sectional study

**DOI:** 10.1371/journal.pone.0247777

**Published:** 2021-03-05

**Authors:** Solomon Ahmed Mohammed, Minilu Girma Yitafr, Birhanu Demeke Workneh, Abel Demerew Hailu

**Affiliations:** 1 Department of Pharmacy, College of Medicine and Health Science, Wollo University, Dessie, Ethiopia; 2 Department of Pharmacy, College of Medicine and Health Science, Debre Berhan University, Debre Berhan, Ethiopia; 3 Department of Pharmacy, Dessie Health Science College, Dessie, Ethiopia; St. Paul’s Hospital Millenium Medical College, ETHIOPIA

## Abstract

**Introduction:**

HIV/AIDS remains a public health concern affecting millions of people across the world. Although the health-related quality of life (HRQoL) of patients living with HIV has significantly improved after treatment, its chronicity makes the HRQoL uncertain. This study assessed factors associated with the health-related quality of life among people living with HIV/AIDS on HAART in North-East Ethiopia.

**Methods:**

An institutional-based cross-sectional study was conducted from March to April 2018, and systematic random sampling was used to select 235 participants who were on HAART. HRQoL was assessed using the Medical Outcomes Study HIV Health Survey. Descriptive and multiple linear regression analysis were computed using the statistical package for social sciences version 20.

**Results:**

The study revealed one-factor structure and had good overall internal consistency (78.5). Over one-third (42.6%; 95% CI; 36.2%, 48.9%) of participants had good HRQoL. The least HRQoL mean score was found for cognitive functioning 32.21(±19.78), followed by social functioning 40.58(±29.8). Factors associated with the overall HRQoL were 25–45 years of age (β = − 3.55, 95% CI;-6.54, -0.55), working in private sector (β = -5.66, 95% CI;-9.43, -1.88), government (β = -4.29, 95% CI;-7.83, -0.75) and self-employment (β = -8.86, 95% CI;-13.50, -4.21), 100–200 (β = − 4.84, 95% CI;-9.04, -0.63) and 201–350 CD4 at the time of diagnosis (β = − 7.45, 95% CI;-11.73, -3.16), 351–500 current CD4 level (β = 8.34, 95% CI;5.55, 11.41), 6–10 years of disease duration (β = -8.28, 95% CI;-12.51, -4.04), WHO stage II (β = -4.78, 95% CI;-8.52, -1.04) and III (β = 3.42, 95% CI;0.06, 6.79) during treatment initiation and not taking of Cotrimoxazole prophylaxis (β = -5.79, 95% CI;-8.34, -3.25).

**Conclusions:**

High proportion of participants had a poor HRQoL. Routine assessment and appropriate interventions at each visit is recommended to improve HRQoL.

## Introduction

Human Immunodeficiency Virus (HIV)/ Acquired Immunodeficiency Syndrome (AIDS) is being a global concern, and people of the world are suffering from this problem. Between 1990 and 2015, 34 million people died from AIDS-related causes, where 15–24 years old people comprised about one-third of the people who acquired HIV infection globally [1]. In 2019, 38 million people are living with HIV and 1.7 million people were newly infected. AIDS-related illnesses were responsible for 690,000 people’s death. Nearly 25.4 million people were accessing antiretroviral therapy [2]. Among the newly HIV infections per day, about 61% were in sub-Saharan Africa. In Ethiopia, 23,000 people were newly infected in 2018 [3].

According to the World Health Organization (WHO), quality of life (QoL) is an individual’s perception of their position in life in the context of the culture and value systems in which they live and concerning their goals, expectations, standards, and concerns [4]. When the quality of life is considered in the context of health and disease, it is referred to as health-related quality of life (HRQoL) [5]. Despite several QoL instruments (short form-36 health survey [6], short form-12 [7] and World Health Organization quality of life (WHOQOL)-BREF [8]) that have been used to evaluate HRQoL of HIV-infected patients, each has a distinct feature. Among these, the Medical Outcomes Study HIV Health Survey (MOS-HIV) allows calculation in both individual scale and summary scores and incorporates three domains (cognitive functioning, health distress, and quality of life) that are not measured by the short form-36 [9].

Health-related quality of life is an important indicator to assess health intervention outcomes, inform patient management, and policy development [5]. Assessing HRQoL become an integral part of treatment follow-up and provides valuable feedback about the effectiveness of therapeutic interventions [10], identify the need for health services improvements, and investigate factors predicting well-being. Since highly active antiretroviral treatment (HAART), HRQoL measurement in HIV/AIDS has advanced rapidly to evaluate the effectiveness of treatment and intervention programs [5].

Various kinds of literature across the world indicated that social problems such as stigma, poverty, depression, substance abuse, and cultural beliefs [11], socio-demographic characteristics [12–14], socioeconomic characteristics [12–15], presence of comorbidities [16], stage of the disease, psychological [17], and clinical factors [13, 14] can affect the HRQoL of patients with HIV/AIDS. Moreover, injection drug use [13], alcohol drinking [11], depression [18], and spiritual belief on their disease and medication had also an effect on the mental and physical health of HRQoL [17, 19]. Although the HRQoL of patients has significantly improved after treatment with HAART [1], drug-related side effects [14], poor adherence to HAART [11], and irregular medical follow-up compromised HRQoL [12].

The HIV/AIDS pandemic is the most important and urgent public health challenge facing governments and civil societies around the world [20]. The psychological, social, and economic burden of the disease associated with its chronicity made the HRQoL of the patients uncertain [11]. The MOS-HIV health survey consisted of a culturally adapted questionnaire [18]. All the items in the general health perception, pain, mental health, and energy dimensions are similar to the generic questionnaire of short form-36, but the rest 6 dimensions were different [6]. HRQoL of people living with HIV remains under-researched in Africa and particularly Ethiopia, yet it is an important health metric; no study was conducted on the HRQoL of adults with HIV from Dessie. Therefore, this study aimed to examine the status of HRQoL and associated factors among people living with HIV/AIDS on HAART in North-East Ethiopia.

## Materials and methods

### Study area and period

This study was conducted from March 2018 to April 2018 at the Dessie Referral Hospital, Dessie town, Amhara National Regional State, North-East Ethiopia. Dessie is located in the South Wollo zone, 401 kilometers away from Addis Ababa. Dessie is one of the oldest cities in the country preferred to medical tourism and a center for trade. Dessie Referral Hospital is serving the populations of Dessie town (219,978) and the surrounding population (7 million). The hospital has different units. During the study period, 6258 peoples living with HIV were on regular follow-up at the HIV comprehensive care clinic.

### Study design

The institutional-based cross-sectional study design was conducted at the HIV comprehensive care clinic of the Dessie Referral Hospital.

### Study population

Participants living with HIV/AIDS on HAART who have periodic regular follow-ups in the ART clinic were the study population.

### Inclusion and exclusion criteria

Participants whose age 18 and greater and who were willing to give consent were included. Patients who were unable to hear, participants who were on HAART for less than 6 months, and those without updated CD4 records were excluded.

### Sample size determination and sampling procedure

The sample size was determined by using a single proportion formula using 73% good HRQoL [21], 95% confidence level, 5% tolerable sampling error, and a 10% non-response rate. There were 6258 registered patients, the sample size was adjusted, and finally, 251 participants were included in this study. On average, the HIV comprehensive care clinic offers services for 70 patients per day. Systematic random sampling was used to select the study participants. The total number of patients who visited the ART comprehensive clinic was taken from the registration book and divided by the total sample size to get the interval. So, the questionnaires were given for every interval. Participants who refused to participate were taken as non-respondents.

### Data collection

Data were collected by face-to-face interviews. Data for the outcome variable was collected by one experienced pharmacist. Clinical variables were collected by reviewing the patient’s medical records. Moreover, socio-demographic characteristics and social support-related variables were the data collected other than the use of the MOS-HIV questionnaire using a face-to-face interview. The principal investigators coordinated data collection.

### Outcome variable

Data were collected using the MOS-HIV questionnaire [4]. The questionnaire was translated into Amharic and then back-translated to English to check message consistency. Two pharmacy professionals from Wollo University did the forward translation from the English language to the Amharic language. Then, the Amharic version of the questionnaire was sent to other pharmacy professionals, who translate it back to the English language. Finally, the researchers compared the original questionnaire to the back-translated questionnaire. The discrepancy was categorized as minor (wording issues) and major (change in the meaning of questionnaire) and resolved through discussion among the two translating groups.

The outcome variable of the study was the HRQoL of people living with HIV/AIDS on HAART. The HRQoL was assessed using the MOS-HIV health survey which contains 35 questions in 10 dimensions, including general health perceptions, physical functioning, role functioning, pain, social functioning, mental health, energy, health distress, cognitive functioning, and quality of life. One additional item assessed health transition. The subscales of the MOS-HIV are scored as summated rating scales on a 0–100, with higher values indicating better HRQoL [22].

Participants rated the extent to which they had experienced each of the 35 items over the past 1 month. The items and scales of the MOS-HIV health survey were scored by item recoding (11 of the 35 items in the survey require recoding item scores), each scale is summed to compute raw scale scores, and raw scale scores are transformed to a 0–100. The scoring was done based on the following formula: Transformation formula = (100/(the top of the range for the sum of the untransformed item scores—the lowest possible score of the untransformed scale)) * (domain raw score—the lowest possible score of the untransformed scale). The physical and mental summary score was calculated based on the 10 scale scores in the MOS-HIV health survey. For each scale, a z-score transformation was calculated, the physical and mental summary scores using the scoring coefficients were computed and, multiplied by the scoring coefficients and aggregated. The final summary score was calculated as: physical health summary score = 50 + (physical health summary * 10) and mental health summary score = 50 + (mental health summary * 10). The total mean scores of the items were calculated and dichotomized into poor versus good HRQoL [22].

### Independent variables

The predictor variables were socio-demographic characteristics of HIV participants, clinical variables, and social support-related variables.

Socio-demographic variables were collected using questionnaires adapted from literature [21, 23] were sex, age, educational status, marital status, working status, and income. Education was categorized based on their level of enrollment in various levels of educational sectors. Understanding the participants’ chances of having some meaningful day-to-day interactions with people, working status was categorized into private, government, self-employed, and unemployed. Social support-related factors (perceived community discrimination and perceived social support) were assessed by asking participants about their perceived community discrimination and perceived social support and were categorized as yes or no.

Clinical variables (duration of disease, CD4 at the time of diagnosis, current CD4 level, duration of a treatment, WHO clinical stage at treatment initiation, current WHO stage, comorbid condition, treatment regimen during initiation, current treatment regimen, taking cotrimoxazole prophylaxis, taking isoniazid prophylaxis, and taking therapeutic food) were collected by reviewing the patient’s medical records.

### Data management and analysis

All data were examined for completeness and consistency before processing. There were no missing data. Data were entered and analyzed using the Statistical Package for Social Sciences version 20.

There are several methods available for the statistical analysis of MOS-HIV data [22]. Cronbach’s alpha was calculated to determine the internal consistency of the MOS-HIV health survey. Reliability was considered to be good if the α value was >0.7.

Validity was analyzed in terms of construct validity. Principal factor analyses were used to test construct validity. Monte Carlo’s principal component analysis for parallel analyses specifying direct Oblimin and Varimax rotation was conducted to test the presence of similar two-factor structures. An eigenvalue of ≥1 was taken as an acceptable factor loading.

After bivariate logistic regression analyses, multiple linear regressions were computed for variables with a p-value less than 0.25, and variables with a p-value less than 0.05 were taken as statistically significant for the association between predictor variables and HRQoL. Results were presented as B-coefficient, 95% Confidence interval (CI), and p-value.

The assumptions of least square regression (random sampling, linearity, normal distribution, constant variance, and multi-colinearity) were checked. A plot of a graph of observed predicted values was symmetrically distributed. In the multiple linear regression model, the correlation matrix showed that there was no multicollinearity between dependent and independent variables. The low variance inflation factor (<2.1) indicated that the associated independent variable has a lower colinearity with the other variables in the model. Homoscedasticity is assessed by visual inspection of plots of residuals versus predicted values. The error term in the regression also had the same variance in the residual predicted values versus the time series plot. The normal probability plot showed that the error term was normally distributed. Kolmogorov-Smirnov (0.20) and Shapiro-Wilk (0.40) tests were non-significant. The Levene statistic (p-value = 0.21) for homogeneity of variance with a p-value greater than 0.05 was considered as a well-fitting regression model, and the percentage of the variability predicted by the model was explained by the R square (61.7%).

The mean score was used to dichotomized the HRQoL score as good and poor. A mean score value of 55 and above was considered as good HRQoL, and below 55 was classified as a poor HRQoL score.

### Ethics approval and consent to participate

The research was approved by the Ethics Review Committee of the Pharmacy Department, Wollo University. Before data collection, pertinent information to study participants was provided. Study participants who participated in the study gave their verbal consent. During the data collection process, the confidentiality of the study participants’ related data was maintained.

## Results

### Socio-demographic, psychosocial, and clinical characteristics of the study participants

The response rate was 93.62%. Sixteen participants were refused to participate in the study. None of the participants were excluded because of unable to hear, less than 6 months of HAART initiation, and without updated CD4 records. Over half of the study participants (57.9%) were female. The majority was in the age range of 25–45 years (54.9%) and completed secondary education (48.5%). Participants having college-level education or above 63.37(5.63) and self-employed 60.08(13.72) had the highest HRQoL mean score and standard deviation (SD). Being unemployed had the lowest HRQoL mean score and SD 46.82(11.40).

A statistically significant HRQoL mean score was found in univariate analysis for age, educational status, work status and marital status of participants. Participants who were 18–24 (β = − 6.48, p < 0.001) and 25–45 years of age (β = − 6.80, p < 0.001) were more likely to have lower HRQoL compared to greater than 45 years of age. Participants who were illiterate (β = 12.40, p < 0.001), attended primary education (β = 12.92, p < 0.001) and secondary education (β = 9.39, p < 0.001) were more likely to have better HRQoL compared to those attended college level education and above. Single participants had a better HRQoL (β = 9.15, p < 0.001) than Windowed. Participants who were working in private sector (β = -9.65, p < 0.001), government (β = -9.70, p < 0.001) and their work (β = -13.26, p < 0.001) were more likely to have lower HRQoL (Table 1).

**Table 1:** Mean HRQoL score across socio-demographic characteristics of participants in Dessie referral hospital, 2018.

Variable		Frequency (%)	Mean (SD)	P-value	β(95%CI)
**Sex**	Female	136 (57.9)	54.47 (12.35)	0.58	0.87 (-2.31,4.07)
	Male	99 (42.1)	55.35 (12.18)	Ref	Ref
**Age**	18–24	50 (21.3)	53.47 (7.26)	0.006	-6.48 (-11.07,-1.89)
	25–45	129 (54.9)	53.15 (13.20)	≤0.001	-6.80 (-10.57,-3.02)
	>45	56 (23.8)	59.95 (12.33)	Ref	Ref
**Educational status**	Illiterate	16 (6.8)	50.96 (8.87)	≤0.001	12.40 (5.89,18.92)
	Primary	57 (24.3)	50.45 (17.23)	≤0.001	12.92 (8.50,17.34)
	Secondary	114 (48.5)	53.98 (9.86)	≤0.001	9.39 (5.51,13.27)
	College and above	48 (20.4)	63.37 (5.63)	Ref	Ref
**Marital status**	Single	90 (38.3)	50.35 (11.65)	0.001	9.15 (4.18,14.13)
	Married	74 (31.5)	59.02 (10.34)	0.85	0.49 (-4.61,5.59)
	Divorced	43 (18.3)	53.98 (10.75)	0.05	5.52 (-0.05,11.11)
	Widowed	28 (11.9)	59.51 (15.73)	Ref	Ref
**Work status**	Private work	93 (39.6)	56.53 (11.72)	≤0.001	-9.65 (-14.00,-5.29)
	Governmental	55 (23.4)	56.47 (9.46)	≤0.001	-9.70 (-13.60,-5.81)
	Self employed	34 (14.5)	60.08 (13.72)	≤0.001	-13.26 (-18.23,-8.29)
	Unemployed	53 (22.6)	46.82 (11.40)	Ref	Ref
**Income**	≤ 1000 birr	48 (20.4)	54.69 (8.39)	0.88	0.35 (-4.53,5.24)
	1001–2000 birr	58 (24.7)	54.44 (18.33)	0.85	0.42 (-4.24,5.09)
	2001–3000 birr	78 (33.2)	54.95 (11.40)	0.96	0.09 (-4.28,4.47)
	> 3000 birr	51 (21.7)	55.05 (7.63)	Ref	Ref

β: Unstandardized coefficient, Ref: Reference group.

Below half (44.2%) of the participants were lived with HIV/AIDS for more than 10 years. Below half (46.8%) of the participants were taking HAART for 6–10 years. Nearly one-third (31.4%) and (14%) had above 500 CD4 during data collection and at the time of diagnosis respectively. Concerning HRQoL mean score, participants with less than the year duration of the disease had the highest (62.84) mean score as compared to prolonged durations.

Participants who had 201–350 CD4 at the time of diagnosis (β = − 6.64, p = 0.01) and 351–500 CD4 during data collection (β = 10.07, p < 0.001) had lower and better HRQoL compared to those who had above 500 CD4 respectively. Participants who had less than year (β = -13.43, p < 0.001), and 6–10 years of disease duration (β = -14.35, p < 0.001) were more likely to have lower HRQoL compared to those had for more than 10 years. Participants who were currently at WHO stage II had a lower HRQoL (β = -18.27, p < 0.001) than WHO stage III. Participants who reported not taking of Cotrimoxazole prophylaxis (β = -9.08, p < 0.001), Isoniazid prophylaxis (β = -3.70, p < 0.02), and Therapeutic food (β = -8.87, p < 0.001) were more likely to have lower HRQoL compared to those taking (Table 2).

**Table 2:** Mean HRQoL score across clinical and psychosocial characteristics of respondents in Dessie referral hospital, 2018.

Variable		Frequency (%)	Mean (SD)	P-value	β(95%CI)
**Duration of disease**	< 1year	10 (4.3)	62.84 (5.45)	0.04	-8.22 (-16.19,-0.25)
	1–5 year	36 (15.3)	53.76 (6.45)	0.72	0.84 (-3.81,5.50)
	6–10 year	85 (36.2)	54.63 (12.98)	0.99	-0.01 (-3.54,3.50)
	> 10 year	104 (44.2)	54.61 (13.44)	Ref	Ref
**CD4 at the time of diagnosis**	≤100	12 (5.1)	51.33 (9.20)	0.83	0.84 (-7.15,8.85)
	100–200	121 (51.5)	53.64 (14.83)	0.53	-1.46 (-6.12,3.20)
	201–350	69 (29.4)	58.83 (9.41)	0.01	-6.64(-11.67,-1.62)
	> 500	33 (14)	52.13 (2.42)	Ref	Ref
**Current CD4 level**	100–200	18 (7.7)	58.45 (15.13)	0.76	-0.84 (-6.42,4.74)
	201–350	19 (8.1)	61.75 (6.86)	0.13	-4.14 (-9.59,1.31)
	351–500	124 (52.8)	57.61 (10.99)	≤0.001	10.07 (6.81,13.32)
	> 500	74 (31.4)	47.54 (11.43)	Ref	Ref
**Duration of treatment**	<1 year	14 (6)	59.93 (6.68)	≤0.001	-13.43 (-20.0,-6.97)
	1–5 year	75 (31.9)	49.06 (9.57)	0.23	-2.56 (-6.78,1.66)
	6–10 year	110 (46.8)	60.86 (10.82)	≤0.001	-14.35 (-18.36,-10.35)
	< 10 year	36 (15.3)	46.50 (12.76)	Ref	Ref
**WHO clinical stage at treatment initiation**	I	5 (2.1)	50.40 (5.21)	0.17	7.19 (-3.29,17.67)
	II	37 (15.7)	61.08 (6.63)	0.12	-3.50 (-7.95,0.95)
	III	103 (43.8)	50.41 (10.33)	≤0.001	7.17 (3.88,10.46)
	VI	90 (38.4)	57.58 (14.36)	Ref	Ref
**Current WHO stage**	I	20 (8.5)	45.47 (11.31)	0.02	-5.50 (-10.41,-0.59)
	II	80 (34)	63.71 (9.31)	≤0.001	-18.24 (-23.36,-13.11)
	III	135 (57.5)	50.97 (10.87)	Ref	Ref
**Comorbid condition**	Diabetes mellitus	10 (4.3)	63.10 (9.27)	0.02	-12.12 (-19.69,-4.55)
	Hypertension	19 (8.1)	48.85 (8.33)	0.59	1.53 (-4.07,7.15)
	Epilepsy	6 (2.6)	49.03 (2.99)	0.08	-8.37 (-18.05,1.29)
	Psychosis	13 (5.5)	63.89 (4.58)	0.03	-10.22 (-16.91,-3.53)
	No comorbidity	187 (79.5)	54.56 (12.75)	Ref	Ref
**Treatment regimen during initiation**	Efavirenz based	111 (47.2)	61.23 (9.87)	≤0.001	-12.11(-14.86,-9.36)
	Nevirapine based	124(52.8)	49.11(11.36)	Ref	Ref
**Current treatment regimen**	Efavirenz based	115 (48.9)	54.54 (11.97)	0.71	0.58(-2.57,3.74)
	Nevirapine based	120(51.1)	55.13(12.58)	Ref	Ref
**Cotrimoxazole prophylaxis**	No	134(57)	58.74(11.67)	≤0.001	-9.08(-12.05,-6.11)
	Yes	101 (43)	49.66 (11.09)	Ref	Ref
**Isoniazid prophylaxis**	No	158(67.2)	56.05(12.36)	0.02	-3.70(-7.03,-0.37)
	Yes	77 (32.8)	52.35 (11.75)	Ref	Ref
**Therapeutic food**	No	171(72.8)	57.25(11.86)	≤0.001	-8.87(-12.23,-5.51)
	Yes	64 (27.2)	48.38 (10.97)	Ref	Ref
**Perceived community discrimination**	No	90(38.3)	52.63(12.43)	0.03	3.57(0.35,6.79)
	Yes	145 (61.7)	56.21 (11.99)	Ref	Ref
**Perceived social support**	No	203(86.4)	54.43(11.75)	0.20	-10.02(-14.44,-5.60)
	Yes	32 (13.6)	57.40 (15.07)	Ref	Ref

β: Unstandardized coefficient, Ref: Reference group.

### HRQoL of participants on HAART

The highest HRQoL mean score was found for physical functioning 91.02(±18.05). Cognitive functioning had the lowest HRQoL 35.21(±19.78). Physical functioning (91.02±18.05) and role functioning **(**88.08±25.03) had the highest HRQoL mean score and SD (Table 3). Over one-third (42.6%; 95% CI; 36.2%, 48.9%) of participants on HAART had a good quality of life.

**Table 3:** HRQoL domains mean score of participants on HAART in Dessie referral hospital, 2018.

QoL domains (number of items)	Mean (SD)	Minimum (Maximum)	Domains of quality of life	
			Poor n (%)	Good n (%)
**General health (5)**	44.51 (16.62)	20 (85)	111 (47.2)	124 (52.8)
**Physical functioning (6)**	91.02 (18.05)	8.33 (99.96)	58 (24.7)	177 (75.3)
**Role functioning (2)**	88.08 (25.03)	0 (100)	48 (20.4)	187 (79.6)
**Social functioning (1)**	40.85 (29.8)	0 (75)	117 (49.8)	118 (50.2)
**Cognitive functioning (4)**	35.21 (19.78)	0 (68.75)	113 (48.1)	122 (51.9)
**Pain (2)**	46.89 (16.11)	22.22 (77.77)	110 (46.8)	125 (53.2)
**Mental health (5)**	47.42 (16.79)	20 (75)	118 (50.2)	117 (49.8)
**Energy/Fatigue (4)**	47.34 (20.14)	18.75 (81.25)	117 (49.8)	118 (50.2
**Health distress (4)**	60.42 (26.83)	6.25 (93.37)	130 (55.3)	105 (44.7)
**Quality of life (1)**	52.23 (21.37)	25 (100)	140 (59.6)	95 (40.4)
**Health transition (1)**	49.25 (23.72)	0 (100)	82 (34.9)	153 (65.1)
**Total**	54.84 (12.26)	32.24 (77.42)	135 (57.4)	100 (42.6)

### Psychometric characteristics of the HRQoL measure

Cronbach’s *α* value was above the standard for all except role functioning (0.34), general health (0.50), and pain (0.48). The Cronbach’s *α* value for the overall scale was 0.78. Cronbach’s alpha was not calculated for sub-domains with 1 item (social function, quality of life, and health transition). Monte Carlo’s principal component analysis of parallel analysis of MOS-HIV sub-scales resulted in the extraction of one factor (general health had Eigenvalue of 3.1). Thus, the analysis of physical or mental health summary scores was not done further.

### Factors associated with overall HRQoL

In the backward multiple linear regression, age, work status, CD4 at time of diagnosis, current CD4 level, duration of treatment, WHO clinical stage at treatment initiation, and taking Cotrimoxazole prophylaxis were found to have statistically significant associations with mean overall HRQoL. Participants who were 25–45 years of age (β = − 3.55, p = 0.02) were significantly more likely to have lower HRQoL compared to those greater than 45 years of age. Participants who were working in private sector (β = -5.66, p = 0.03), government (β = -4.29, p = 0.01) and their work (β = -8.86, p < 0.001) were more likely to have lower HRQoL than unemployed. Participants who had 100–200 (β = − 4.84, p = 0.02) and 201–350 CD4 at the time of diagnosis (β = − 7.45, p < 0.001) and 351–500 CD4 during data collection (β = 8.34, p < 0.001) had lower and higher HRQoL compared to those who had above 500 CD4 respectively. Participants who had 6–10 years of disease duration (β = -8.28, p < 0.001) were more likely to have lower HRQoL compared to those who had for more than 10 years. Participants who were at WHO stage II (β = -4.78, p = 0.01) and III (β = 3.42, p = 0.04) during treatment initiation had a lower HRQoL and higher than WHO stage III participants. Participants who reported not taking Cotrimoxazole prophylaxis (β = -5.79, p < 0.001) were more likely to have lower HRQoL compared to those who take (Table 4).

**Table 4:** Factors associated with mean overall HRQoL of participants on HAART in Dessie referral hospital, 2018.

Variable		P-value	Β	95%CI
Age	18–24	0.46	-1.52	-5.63,2.57
	25–45	0.02	-3.55	-6.54,-0.55
	>45		Ref	Ref
Work status	Private work	0.03	-5.66	-9.43,-1.88
	Governmental	0.01	-4.29	-7.83,-0.75
	Self employed	≤0.001	-8.86	-13.50,-4.21
	Unemployed		Ref	Ref
CD4 at the time of diagnosis	≤100	0.41	2.78	-3.97,9.54
	100–200	0.02	-4.84	-9.04,-0.63
	201–350	0.001	-7.45	-11.73,-3.16
	> 500		Ref	Ref
Current CD4 level	100–200	0.27	2.58	-2.08,7.24
	201–350	0.06	-4.24	-8.77,0.28
	351–500	≤0.001	8.34	5.55,11.14
	> 500		Ref	Ref
Duration of treatment	<1 year	0.41	-2.57	-8.72,3.58
	1–5 year	0.38	1.70	-2.16,5.57
	6–10 year	≤0.001	-8.28	-12.51,-4.04
	< 10 year		Ref	Ref
WHO clinical stage at treatment initiation	I	0.22	4.95	-3.02,12.93
	II	0.01	-4.78	-8.52,-1.04
	III	0.04	3.42	0.06,6.79
	VI		Ref	Ref
Cotrimoxazole prophylaxis	No	≤0.001	-5.79	-8.34,-3.25
	Yes		Ref	Ref

β is unadjusted, n = 235, R square = 61.7%.

## Discussion

This study assessed the HRQoL and associated factors among people living with HIV/AIDS on HAART in North-East Ethiopia using a unidimensional HIV specific measure with acceptable internal consistency reliability. Age, work status, CD4 at time of diagnosis, current CD4 level, duration of treatment, WHO clinical stage at treatment initiation, and taking Cotrimoxazole prophylaxis were associated with HRQoL among people living with HIV/AIDS on HAART were associated with HRQoL. The present study revealed that close to half of the participants (43%) had a good QoL.

In this study, we observed that close to half of the participants (43%) had a good QoL. This finding compares with previous findings involving HIV patients on treatment from Ethiopia [23, 24] and Togo [25]. The proportion of study participants with good QoL was even higher (>70%) according to other studies from sub-Saharan Africa [26]. However, other studies also reported much lower proportions of participants with good QoL than what we observed [25]. The difference might be due to the variation in the tools used to assess HRQoL among studies and differences in the composition of study subjects.

Various studies reported that the psychological domain (5.99) [27], general health functioning (41.35±10.28) [28], role emotional (41.5±15.1) [29], environment domain (12.9±2.5) [30], and energy (60.6±23.3) [18] had the lowest HRQoL mean score. In Iran, HIV patients had low QoL in all health-related domains [31]. Patients on HAART had also a poorer quality of life in all health-related domains than the general population in the United Kingdom [5]. In this study, the cognitive functioning domain of HRQoL had the lowest mean score of 35.21(±19.78). The possible explanation for this difference might be due to variation in the tools used to assess QoL and differences in the composition of study participants.

The present study revealed that educational status was not significantly associated with HRQoL. This finding discord with other studies conducted elsewhere in the world. Education was significantly correlated with all domains of HRQoL scores [30], and lower education was responsible for lower HRQoL [13, 25, 26]. Thus, it is not surprising that better educational status will yield good HRQoL as patients who have formal education might have superior knowledge regarding the disease, related opportunistic infections, and the medication, and become adherent [32]. The discrepancy might be attributed to the differences in the study areas and periods, and socio-demographic characteristics across the study populations.

Despite marital status promotes health and longevity, this study found a non-significant association. This is because married individuals may have better social support from their partner and children, while widowed and divorced patients may feel sad and discomfort because of the loss of beloved ones and may feel hopeless. This finding contradicted the studies reporting significant associations [26, 28, 33]. The discrepancy might be attributed to the differences in the study populations. The sex of respondents was also not associated with HRQoL. Even if this was in line with the result of a study done in Ethiopia [26], significant associations were also reported [29, 32, 34]. Differences in the study populations might be the reason for the discrepancy.

Participants who were 25–45 years of age (β = − 3.55, p = 0.02) were more likely to have lower HRQoL compared to those greater than 45 years of age. Similarly, the age of participants was significantly associated with psychological health [34] and physical functioning [33]. Upon increasing age, HIV patients are more likely to have a comorbidity, opportunistic infections, and non-adherent to the treatment, which cumulatively reduces their HRQoL [35]. Although the effect of comorbidity on HRQoL was non-significant in this study, having comorbidity was found to be an independent predictor of poor QoL [16, 18, 36, 37]. This is due to the differences in the socio-demographic characteristics of study populations.

In this study, social support had a non-statistically significant effect on HRQoL. However, existing data suggested that social support systems were important predictors of QoL [18, 36, 38] where lack of support from family and good social support were found to be independent predictors of poor [37] and better HRQoL [23, 39]. This might be attributed to the differences in the study populations. Social support will improve the ability to face stress-related health-disease processes, promote health and therapeutic compliance. Better social support will protect against the future enacted and internalized stigma [40].

Although community discrimination does not have a statistically significant effect on HRQoL, HIV patients might treat unjustly and become depressed [41]. Except for the physical domain, a higher level of HIV related discrimination was associated with a lower HRQoL [42]. The discrepancy might be attributed to the differences in the study area. The community members unfairly and unjustly treat patients based on the myth of causal transmission of HIV and preexisting attitudes. People living with HIV might internalize the discrimination they experience, which seriously affects their lives [43].

This study revealed a significant positive association of cotrimoxazole preventive therapy on the overall HRQoL of HIV participants. Preventive therapy was also reported to affect (β = +8.381, p<0.05) on physical health and symptoms [44]. Cotrimoxazole is a safe and inexpensive antibiotic used to treat opportunistic infections. It can help to reduce the high early mortality and morbidity rate of HIV infection regardless of HAART status [45].

Given the longevity achievable with current HAART for persons with HIV infection [5, 36], treatment with HAART was associated with better HRQoL [23, 25]. Longer treatment duration was associated with higher HRQoL domains, except for the spiritual and level of independence domains [46], while all dimensions of QoL have significantly improved [1]. However, second-line antiretroviral therapy has been reported to reduce QoL [44]. This might be attributed to the increased pill burden, side effects of medication, and disease progression. Studies also revealed that the advancement of disease was associated with low HRQoL [42]. The present study was in line with these findings where participants who had 6–10 years of disease duration (β = -8.28, p < 0.001) were more likely to have lower HRQoL compared to those who had for more than 10 years. This was due to the fact that patients who spent longer after knowing their HIV status might be reluctant about the disease, medication, and lifestyle.

The present study revealed that participants who had current 351–500 CD4 (β = 8.34, p < 0.001) had higher HRQoL compared to those who had above 500 CD4 respectively. Despite the non-significant association between the level of CD4 and HRQoL was reported in Ireland [18] and Ethiopia [34, 42], a lower CD4 count was revealed as a factor responsible for lower HRQoL [13, 28, 32]. In Ethiopia, the determination of patient CD4 levels will be done every six months. CD4 cells are a strong marker for disease progression, and the possible explanations might be that nearly half (52.8%) of participants had 351 to 500 CD4 count and patients might have initiated HAART at the advanced WHO clinical stage.

The present study limitations were recall bias and social desirability bias as participants were requested to respond based on their life experience in the past one month before the interview, and the cross-sectional nature of the study made them unable to establish cause-and-effect relationships. Besides, the lack of rigorous validation or cultural adaptation of the MOS-HIV in Ethiopia might make it difficult in understanding the questionnaire.

## Conclusions

In this study, the HRQoL of many Ethiopian adults living with HIV on HAART from the Dessie area was poor. The lowest mean HRQoL scores were observed in the cognitive functioning domain. Age, work status, CD4 at time of diagnosis, current CD4 level, duration of treatment, WHO clinical stage at treatment initiation, and taking Cotrimoxazole prophylaxis were associated with HRQoL among people living with HIV/AIDS on HAART. Routine assessment and appropriate interventions at each clinic visit will improve the HRQoL of people living with HIV/AIDS.

## Supporting information

S1 File Psychometric characteristics of the HRQoL life measure. (DOCX)

## List of abbreviations

AIDS: Acquired immunodeficiency syndrome
ART: Antiretroviral treatment
HAART: Highly active antiretroviral drugs
HIV: Human immunodeficiency virus
HRQoL: Health-related quality of life
MOS: Medical Outcome Study
QoL: Quality of life
WHO: World Health Organization
