# Supplementary material for: Health-related quality of life and associated factors among people living with human immunodeficiency virus on highly active antiretroviral therapy in North East Ethiopia: Cross-sectional study
Source: PLoS One. 2021 Mar 5;16(3):e0247777. doi: 10.1371/journal.pone.0247777 (PMC7935299; doi:10.1371/journal.pone.0247777)
Supplement: S1 File — (DOCX) [file pone.0247777.s001.docx]

**S1 File. Psychometric characteristics of the HRQoL life measure**

The reliability estimates of scale were all acceptable except role functioning (0.34), general health (0.50), and pain (0.48). The Cronbach’s 𝛼 value for the overall scale was 0.78 (Supplementary Table 1). Several coefficients for the correlation between each item and its related scales were low (Supplementary Table 2).

Supplementary Table 1: Reliability of the MOS-HIV sub-scales

| QoL domains | Number of items | Cronbach 𝛼 |
| --- | --- | --- |
| General health | 5 | 0.50 |
| Physical functioning | 6 | 0.86 |
| Role functioning | 2 | 0.34 |
| Social functioning | 1 | NA |
| Cognitive functioning | 4 | 0.83 |
| Pain | 2 | 0.48 |
| Mental health | 4 | 0.84 |
| Energy/Fatigue | 4 | 0.88 |
| Health distress | 4 | 0.89 |
| Quality of life | 1 | NA |
| Health transition | 1 | NA |
| Total |  | 0.78 |

NA: Cronbach’s alpha was not calculated for sub-domains with 1 item

Supplementary Table 2: Inter-correlations of the MOS-HIV scales

| Scales | Mental health | Physical function | General health | Role functioning | Social functioning | Energy | Health distress | Cognitive functioning | Quality of life | Health transition | Pain |
| --- | --- | --- | --- | --- | --- | --- | --- | --- | --- | --- | --- |
| Mental health | 1.00 |  |  |  |  |  |  |  |  |  |  |
| Physical function | -0.34 | 1.00 |  |  |  |  |  |  |  |  |  |
| General health | -0.16 | 0.29 | 1.00 |  |  |  |  |  |  |  |  |
| Role functioning | -0.10 | 0.63 | 0.00 | 1.00 |  |  |  |  |  |  |  |
| Social functioning | 0.83 | -0.40 | -0.39 | -0.16 | 1.00 |  |  |  |  |  |  |
| Energy | 0.91 | -0.43 | -0.35 | -0.15 | 0.86 | 1.00 |  |  |  |  |  |
| Health distress | 0.88 | -0.47 | -0.14 | -0.21 | 0.82 | 0.84 | 1.00 |  |  |  |  |
| Cognitive functioning | 0.61 | -0.30 | -0.01 | -0.24 | 0.53 | 0.56 | 0.74 | 1.00 |  |  |  |
| Quality of life | 0.50 | -0.35 | -0.13 | -0.28 | 0.43 | 0.43 | 0.52 | 0.69 | 1.00 |  |  |
| Health transition | 0.74 | -0.46 | -0.28 | -0.26 | 0.68 | 0.78 | 0.79 | 0.69 | 0.58 | 1.00 |  |
| Pain | 0.71 | -0.27 | -0.27 | -0.04 | 0.64 | 0.78 | 0.59 | 0.39 | 0.28 | 0.53 | 1.00 |

Principal components analysis with direct Oblimin rotation was conducted to test the presence of a similar two-factor structure. The correlation between the two components was low. We found very similar solutions from either of the types of rotations. Monte Carlo's principal component analysis of parallel analysis of MOS-HIV sub-scales resulted in the extraction of one factor from ten scales where health distress and energy loaded most strongly onto the mental health scale (Supplementary Table 3).

Supplementary Table 3: Standardized estimates of factor loading and score of the MOS-HIV scales

| Scales | Component score coefficient | Component matrix |
| --- | --- | --- |
| Mental health | 0.14 | 0.90 |
| Physical function | -0.09 | -0.56 |
| General health | -0.05 | -0.32 |
| Role functioning | -0.05 | -0.30 |
| Social functioning | 0.14 | 0.87 |
| Energy | 0.15 | 0.92 |
| Health distress | 0.15 | 0.92 |
| Cognitive functioning | 0.12 | 0.75 |
| Quality of life | 0.10 | 0.64 |
| Health transition | 0.14 | 0.87 |
| Pain | 0.11 | 0.72 |

Supplementary Figure 1 below presents these the scree plot and the fit indices of MOS-HIV sub-scales. In terms of Eigenvalue, all except one scale had an acceptable factor loading (≥1). These patterns of results suggest the presence of one factor (Supplementary Figure 1).

Supplementary Figure 1: Screen plot for the MOS-HIV scales fit indices
